# Lumbar Magnetic Resonance Imaging Shows Sex-Specific Alterations During Musculoskeletal Aging—A Radio-Anatomic Investigation Involving 202 Individuals

**DOI:** 10.3390/jcm13237233

**Published:** 2024-11-28

**Authors:** Horst Balling, Boris Michael Holzapfel, Wolfgang Böcker, Dominic Simon, Paul Reidler, Joerg Arnholdt

**Affiliations:** 1 Department of Orthopaedics and Trauma Surgery, Musculoskeletal University Center Munich (MUM), University Hospital, Ludwig-Maximilians-Universität Munich, Marchioninistr. 15, 81377 Munich, Germanywolfgang.boecker@med.uni-muenchen.de (W.B.); dominic.simon@med.uni-muenchen.de (D.S.); joerg.arnholdt@med.uni-muenchen.de (J.A.); 2 Center for Spine Surgery, Neckar-Odenwald-Kliniken gGmbH Buchen, Dr.-Konrad-Adenauer-Str. 37, 74722 Buchen, Germany; 3 Department of Radiology, University Hospital, Ludwig-Maximilians-Universität Munich, Marchioninistr. 15, 81377 Munich, Germany; paul.reidler@med.uni-muenchen.de

**Keywords:** aging, brightness, cross-sectional area, lumbar MRI, musculoskeletal, posterior paravertebral muscle, psoas muscle, sex-specific, signal intensity

## Abstract

**Background/Objectives**: Musculoskeletal aging can clinically hardly be distinguished from degenerative disease, especially if symptoms are nonspecific, like lower back pain and reduced physical resilience. However, age-related changes are considered to be physiological until they cause osteoporotic fractures or sarcopenia-related restrictions. This radio-anatomic investigation examines whether findings in lumbar magnetic resonance imaging (MRI) mirror age- and sex-related musculoskeletal differences that help to identify the onset of sarcopenia. **Methods**: Lumbar MRI investigations from 101 women and 101 men were retrospectively evaluated for vertebral and muscular cross-sectional diameter sizes and T2-signal intensities (“T2-brightness”) in axial sections in the L5-level. The results were correlated with the individual’s age to find specific alterations that were indicative of sarcopenia or attributable to the aging process. **Results**: In women (average age 62.6 (34–85) years), musculoskeletal cross-sectional area sizes and diameters were significantly smaller (*p* < 0.00001) compared to those in men (average age 57.0 (21–90) years). The most pronounced structural age-related change was the increasing mean posterior paravertebral muscle brightness (MPPVB), which exceeded the mean vertebral brightness (MVB) earlier and to a greater extent in women than in men (*p* < 0.00001). The brightness difference (∆MVB − MPPVB) was found to indicate (pre-)sarcopenia at values below 25. **Conclusions**: Significant age-related deterioration in muscle quantity and quality was more obvious in women, correlated with the onset of menopause, and progressed to lower levels during aging.

## 1. Introduction

Intersexual musculoskeletal differences are well recognized in the contemporary medical (and especially orthopedic) literature [1,2,3]. These are due to a slightly different gender-specific metabolism that promotes a non-uniform development during musculoskeletal aging [4]. At higher ages, women are frequently affected by considerably reduced bone and muscle mass, leading to imminent health-threatening complications comprising osteoporotic fractures and sarcopenia-related restrictions that limit activities and mobility after menopause [3]. Men do also go through changes, but these generally implicate a less significant loss of bone and muscle mass [3,5]. Today, pre-manifest osteoporosis can be quantified through bone mineral density measurements from lumbar or femoral dual-energy X-ray absorptiometry (DXA) or quantitative computed tomography (QCT) [6,7]. Biochemical bone turnover markers (e.g., ß-crosslaps) and osteometabolic hormone level tests reveal imbalances in bone anabolism and catabolism during anti-osteoporotic therapy [5,8]. Although these diagnostic tools are very specific to diagnose, quantify, and monitor osteoporosis, they are not informative about the associated muscle atrophy. Sarcopenia is defined as a progressive generalized (usually age-related) loss of skeletal muscle mass and strength and is generally diagnosed via questionnaires, clinical tests—such as the grip strength test, the gait speed test, or the timed “up-and-go” test—or with whole-body and appendicular skeletal muscle mass determination, and bioelectrical impedance analysis [9]. However, clinical tests are subjected to bias through painful disability, demotivation, depressive impairment, or co-morbidity-related episodic disturbances, and technical quantification of the extent of osteoporosis and sarcopenia is expensive and resource-consuming.

Previous attempts to compare bone mineral density with findings from lumbar magnetic resonance imaging (MRI) have already shown positive correlations [10,11]. Moreover, if relevant information to characterize a person’s musculoskeletal status could be obtained from the opportunistic evaluation of already existing imaging investigations, a faster clinical workflow and resource-saving effect could result, especially for therapists concerned with geriatric patients.

The current radio-anatomic study on lumbar MRI investigations was performed to basically examine sex-specific differences in musculoskeletal determinants in an inferior lumbar level and to discern whether simple structural measurements are indicative of gender- and age-related musculoskeletal alterations. In particular, we hypothesized that in MRI-based T2-weighted cross-sectional measurements, the posterior paravertebral muscle shows faster age-related fatty degeneration than other musculoskeletal structures, and that sex-related comparisons might help to identify sarcopenic transformation through the definition of a measurable sarcopenia-indicating threshold value.

## 2. Materials and Methods

Radiologic datasets of a consecutive series of individuals who presented to a general hospital from 2 March 2022 to 10 June 2022 were looked through for recent or previous lumbar MRI studies. Imaging material in DICOM format from the hospital’s Picture Archiving and Communication System (PACS) server had to fulfill the inclusion and exclusion criteria given in Table 1.

**Figure 1:**
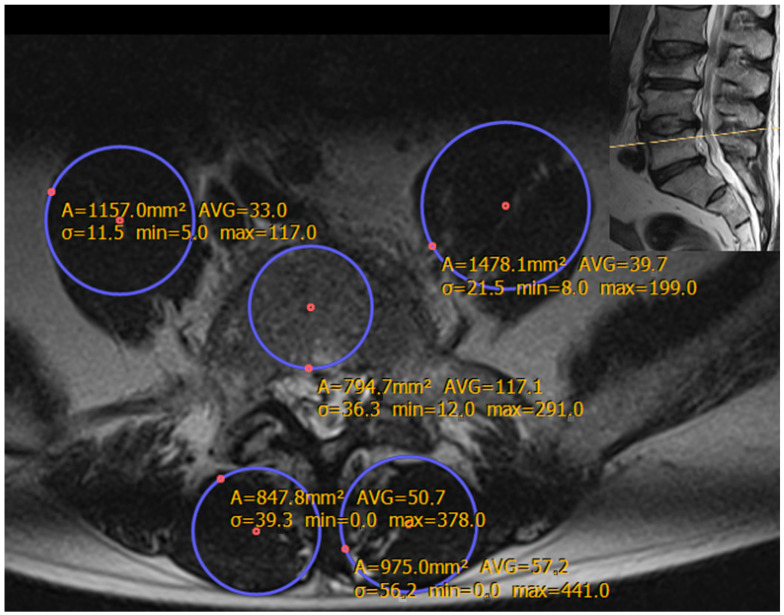
Example showing LIC area size and brightness parameters measured in the vertebral body L5, in both psoas muscles and in both posterior paravertebral muscles. Axial MRI slice in the L5-level of a 59-year-old man with a history of multiple pathologic thoracic and lumbar vertebral fractures. LIC indicates largest inscribed circle; L5, lumbar vertebra V; MRI, magnetic resonance imaging; A, area; AVG, average gray level (signal intensity, or brightness); σ, standard deviation; min, minimum signal intensity, or minimum brightness value; max, maximum signal intensity, or maximum brightness value.

Suitable examinations were collected until a database comprising 101 women and 101 men had been created. Measurements were exclusively conducted in T2-weighted axial lumbar MRI slices depicting the upper half of the fifth lumbar vertebral body in order to create uniform conditions for standardized measurements. To keep these measurements technically simple, the geometric shape of a circle was elected as the best fit to mimic the cross-sectional representation of the fifth lumbar vertebra (LV5), both psoas muscles (PMs), and both posterior paravertebral muscles (PPVMs) (Figure 1).

With the PACS software (RVC Clinical PACS^®^, version 22.2.1.14467, Freiburg, Germany), circular area size and average signal intensity (i.e., T2-brightness) values were calculated from investigated regions of interest (ROIs). In order to avoid influences from neighboring tissues, only largest inscribed circles (LICs) were chosen for evaluation, defined through the following criteria:

1. The outline of the LIC does not cross anatomic borders of evaluated musculoskeletal structures.
2. The outline of the LIC tangentially touches the anatomic confines of evaluated structures in at least two different points.

Thus, in each individual, five circles were drawn to define five ROIs, each of them delivering values for ROI area size and average T2-brightness inside these ROIs. Based on the ROI area size values, we calculated the ROI diameter and considered the transverse diameters in PM-related ROIs as a surrogate for the actual PM size, and the anteroposterior (longitudinal) diameters in LV5-related ROIs and PPVM-related ROIs as surrogates for the actual vertebra and PPVM sizes, respectively. Mean vertebral (MVB), psoas (MPB), and posterior paravertebral muscle brightness (MPPVB) were correlated to age to identify parameters in LV5, PMs, and PPVMs attributable to aging.

### Statistical Assessment

Values were expressed as means and range of continuous variables, or percentages of categorical variables. Sex-specific comparisons were conducted regarding average LIC longitudinal and transverse diameters and mean signal intensities in corresponding ROIs of investigated musculoskeletal structures. Results were displayed as means, ranges, and 95% confidence intervals (CIs). Student’s *t*-test was used to compare normally distributed continuous variables. The significance level was set at 5%. To be able to discern gender-related differences between investigated parameters, the study was planned to detect a difference in MPPVB between women and men of at least 55 points given a standard deviation of a maximum of 100 points within groups. Under these conditions, the desired statistical power of 95% required a sample size of 86 participants per study group calculated with OpenEpi software (Version 3.01, www.openepi.com, accessed on 17 September 2020). Analyses were performed with SPSS 15.0.1 for Windows (SPSS Inc., Chicago, IL, USA).

The MVB values were interpreted as a parameter inversely correlating to bone quality, with increasing T2-brightness indicating decreasing bone density. As the age-related fatty involution of tissues was hypothesized to proceed faster in PPVMs than in the other investigated structures, the difference between MVB and MPPVB (∆MVB − MPPVB) was deemed an indicator for (physiological) age-related muscle degradation. A relevant loss of muscular resilience was supposed to be reached as soon as ∆MVB − MPPVB values would fall below a threshold of 25. The chosen threshold was derived from a study evaluating routine diagnostic lumbar MRI scans of 40 women and 40 men, aged 20–40 years, without structural pathologies of the spine [12]. The overall mean ∆MVB − MPPVB calculated from raw data of this study was 39.2 with a standard deviation of 53.5, and the 95% CI was [27.5, 50.9]. This suggested that ∆MVB − MPPVB values below 25 could be assumed to indicate reduced muscular resilience or (pre-)manifest sarcopenia.

The ratio between corresponding MPPVB and MPB values (MPPVB/MPB ratio) served to show which of the investigated muscle groups degenerated faster during aging. Values above “1” indicated faster PPVM degeneration; those below “1” indicated faster PM degeneration.

Clustered mean ∆MVB − MPPVB values and MPPVB/MPB ratios from every 20 individuals of similar age were analyzed to obtain outlier-adjusted data on age-related muscle quality deterioration. Finally, means of additive ∆MVB − MPPVB values and MPPVB/MPB ratios stepwise from the two youngest (N = 2) to the entire group of men (n = 101) and women (n = 101) were investigated for influences from single additional elderly participants on the group of, respectively, younger individuals.

This retrospective observational study was registered on 30 March 2021 (German Clinical Trials Register, DRKS00024942). Ethical approval was obtained on 18 March 2024 (Ref.-Nr. 23-0866) after data collection.

## 3. Results

In total, 101 women and 101 men (average age 62.6 (34–85) years vs. 57.0 (21–90) years, *p* ≈ 0.009) were enrolled in this investigation during the recruitment period. Lumbar MRI studies suitable for musculoskeletal measurements were available from all participants. Several spinal pathologies were found at similar frequencies for both sexes (Table 2). Only fractures (*p* ≈ 0.002) and spondylolistheses (*p* ≈ 0.02) were significantly more often found in women, whereas “no relevant spinal alterations”, i.e., none of the mentioned pathologies in Table 2, were more prevalent in men (*p* ≈ 0.048).

### 3.1. Sex-Specific Musculoskeletal Analysis (Table 3, Figure 2)

LIC area size and diameter values for LV5, PMs, and PPVMs were significantly higher in men (*p* < 0.00001). MVB and MPB values were insignificantly higher in women (*p* ≈ 0.14 and *p* ≈ 0.61, respectively). Only MPPVB values and MPPVB/MPB ratios were significantly higher in women than in men (*p* < 0.00001, each), whereas ∆MVB − MPPVB values were significantly lower in women (*p* ≈ 0.002).

The supplementary raw data table can be found in the Supplementary Materials.

### 3.2. Age-Related Musculoskeletal Analysis

**Figure 3:**
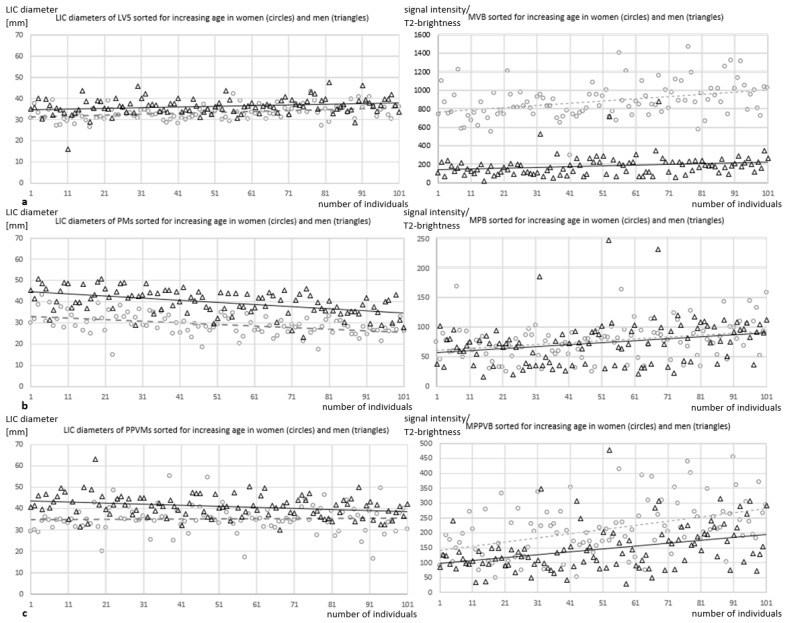
(**a**–**c**) In 101 men (triangles), values of mean LIC diameters in LV5s (**a**), PMs (**b**), and PPVMs (**c**) were significantly higher, and MPPVB values (**c**) were significantly lower (*p* < 0.00001, each) than in 101 women (circles). The dashed and non-dashed lines are trendlines relating to the collectives of women and men, respectively. In older individuals, a distinct decrease in PM diameter and increase in MPPVB were found compared to younger individuals. LIC indicates largest inscribed circle; LV5, lumbar vertebra V; PM, psoas muscle; PPVM, posterior paravertebral muscle; mm, millimeters; MVB, mean vertebral (body) brightness; MPB, mean psoas (muscle) brightness; MPPVB, mean posterior paravertebral (muscle) brightness.

In older individuals of any gender, MPPVB values reached higher levels than respective MPB values and constituted the most considerable structural changes during aging (Figure 3).

Clustered mean ∆MVB − MPPVB values from every 20 individuals of similar age showed an age-related relative decrease with an earlier onset of negative values in women compared to men at a mean age of 56.0 years (N = 49) versus 69.9 years (N = 90), respectively, and with negative values throughout in female subgroups aged 66.9 years and older (N ≥ 66).

**Figure 4:**
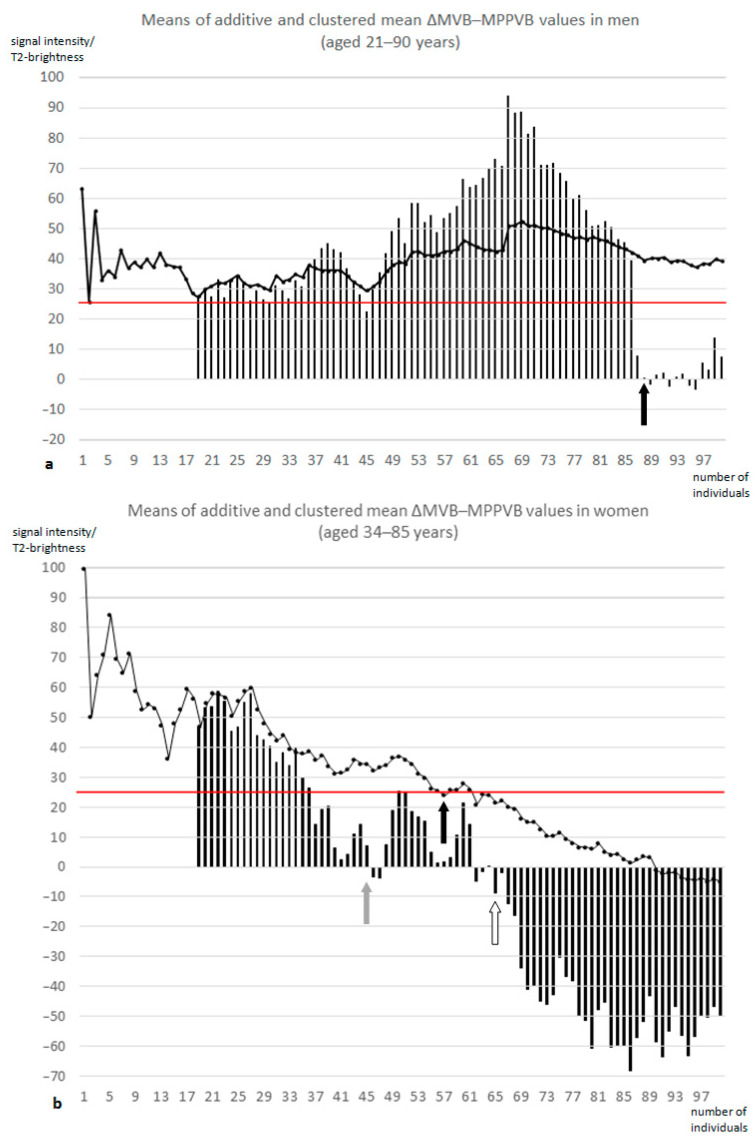
(**a**,**b**) In 101 men (**a**), means of additive ∆MVB − MPPVB values (black curve) varied around the mean of 39.3 points without crossing the threshold of 25 points (red line), defined to indicate reduced musculoskeletal resilience, with positive values in subgroups of 20 men sorted for increasing age until the 70th subgroup (columns, black arrow). In 101 women (**b**), means of additive ∆MVB − MPPVB values (black curve) continually decreased and fell below (black arrow) the threshold of 25 points (red line), with positive values in subgroups of 20 consecutive women sorted for increasing age until the 27th subgroup (gray arrow) and throughout negative values after the 46th subgroup (white arrow). ∆MVB − MPPVB indicates difference between mean vertebral brightness and mean posterior paravertebral (muscle) brightness.

In men, means of additive ∆MVB − MPPVB values ranged from 25.6 to 63.5 without detectable influences of single elderly individuals on respective younger collectives (Figure 4a).

In women, these values ranged from 99.7 in individuals younger than 34 years to −5.1 in the entire female collective. Values below the threshold of 25 were defined to indicate (pre-)sarcopenic muscle degradation as soon as women over 70 years were included (N ≥ 63) (Figure 4b).

**Figure 5:**
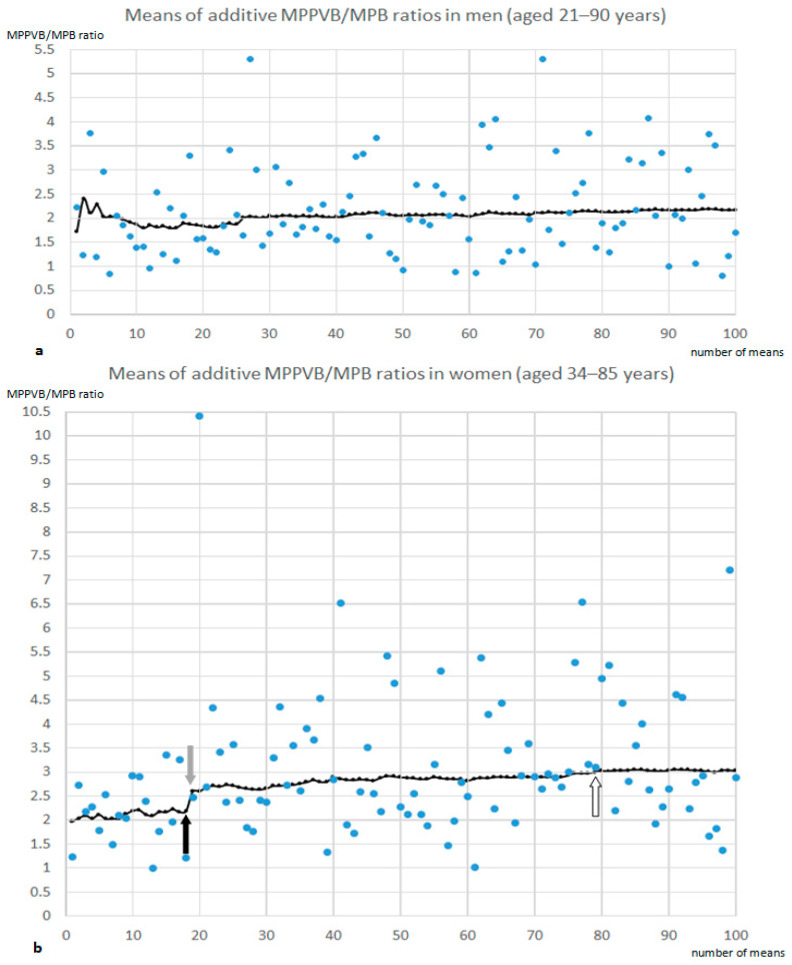
(**a**,**b**) In 101 men (**a**), means of additive MPPVB/MPB ratios (black curve) varied between 1.73 and 2.40 with a slightly increasing tendency at higher ages. In 101 women (**b**), means of additive MPPVB/MPB ratios (black curve) showed a level rise from values below 2.2 (in groups with women aged 47 years and younger, black arrow) to values above 2.5 (in groups with women aged 47 years and older, gray arrow), exceeding a value of 3.0 in groups containing women older than 79 years (white arrow). MPPVB indicates mean posterior paravertebral (muscle) brightness; MPB, mean psoas (muscle) brightness.

Means of additive MPPVB/MPB ratios were almost constant in participating men at any age (Figure 5a). In women, however, a level change was found between younger (N ≤ 19) and older collectives as soon as 47-year-old women were included (Figure 5b).

## 4. Discussion

This is the first study in the literature that investigates musculoskeletal parameters, including signal intensity, in T2-weighted axial images of lumbar MRI studies for their relationship to sex-specific differences during aging. Baseline conditions with a threefold higher prevalence of (pathologic) fractures in participating women (*p* ≈ 0.002, Table 2) are attributable to an increased susceptibility to osteoporotic fractures in the subgroup of postmenopausal women. The higher prevalence of spinal instability (spondylolisthesis) in women (*p* ≈ 0.02) might be due to sex-specific differences in connective tissue strength. The twofold higher occurrence of “no relevant spinal alterations” in men (*p* ≈ 0.048) could be due to the significantly lower average age of male participants in this study (*p* ≈ 0.009).

Huang et al. showed that vertebral fractures in postmenopausal women with underlying osteoporosis or reduced bone mass were associated with fewer paraspinal and PM volumes [13]. However, their conclusions were based on a small number of participants (n = 24). Shayganfar et al. correlated MRI-based data to DXA-based T-scores in 82 postmenopausal and 69 healthy young women and could identify patients at risk of osteoporosis with high sensitivity and specificity using a newly developed MRI-based score [14]. Another modern approach to quantifying vertebral compressive strength is to create a bone model to which loads are applied analytically. Such a finite element model was shown to correlate better with vertebral compressive strength than QCT [15].

An osteoporosis-associated phenomenon in the elderly, especially in women, is muscle mass loss, which results in sarcopenia. Unlike osteoporosis, sarcopenia requires a different approach to be accurately visualized. Most authors recommend DXA, computed tomography, or MRI in order to study body composition, which implies the identification of quantitative and qualitative changes in muscle mass [16,17]. A systematic review of MRI-derived sarcopenia-related biomarkers could show that muscle cross-sectional area was mostly used for muscle quantity estimation and that muscle fat content or fiber architecture rather served to assess muscle quality [18]. However, besides the distinct advantages of MRI for investigating body composition, standardized assessment protocols and diagnostic MRI-based cut-off values have not been established yet [17].

Other authors have performed CT-based body composition measurements in the L3-level [19,20] or in the psoas and mid-thigh muscle [21], which led to a variety of proposed cut-off values for diagnosing sarcopenia. In the current investigation, measurements have been performed in transsectional T2-weighted MRI slices in the L5-level. PMs and PPVMs were investigated using the circle-measuring tool of a commonly available clinical PACS software (version 22.2.1.14467). Investigated ROIs were defined by the largest fitting inscribed circle in order to keep the complexity of data acquisition at an acceptable level, which is important for successfully inaugurating new applications in clinical settings.

### 4.1. Sex-Specific Analysis

Significantly higher mean LIC area size and diameter values of LV5, PMs, and PPVMs in men (*p* < 0.00001) suggest that these parameters are generally associated with body height, although such correlations were not explicitly investigated.

In women, significantly higher MPPVB values (*p* < 0.00001) and insignificantly higher MVB (*p* ≈ 0.14) and MPB values (*p* ≈ 0.61) indicated faster age-related musculoskeletal degeneration and faster loss of muscle strength in PPVMs compared to PMs.

The finding of constant signal intensities in PMs in all participants (Figure 3b) contradicts reports in the literature where PM parameters were proposed to be clinically useful for diagnosing sarcopenia, especially in individuals with liver cirrhosis [22] or after colorectal cancer surgery [23]. Contrary to these reports, Baracos called the assumption that PM parameters were indicative of sarcopenia a flawed premise [24]. However, cited studies reported CT-based findings, whereas the current investigation evaluated MRI-based data that support Baracos’s arguments questioning the usefulness of PM parameters for detecting or confirming sarcopenia.

### 4.2. Age-Related Analysis

During aging, MVB and MPB values varied within almost constant limits in both sexes, whereas MPPVB values appeared to increase. This effect was more evident in women, especially if individual ∆MVB − MPPVB values were investigated (Figure 4), which could relate to the higher prevalence of osteoporosis and sarcopenia in postmenopausal women. In men, means of additive ∆MVB − MPPVB values were almost constant around a value of 40 (Figure 4a). In women, this parameter followed an ever-descending curve until the predefined value of 25, indicating reduced muscular resilience, was reached for the first time in the subgroup of 20 women with an average age of 51.3 years and stayed constantly below this threshold as soon as over-70-year-old women were included (Figure 4b). These findings are in line with recommendations of the US Preventive Services Task Force to perform bone measurement testing for osteoporosis screening in women aged 65 years and older [25].

In men sorted for increasing age, means of additive MPPVB/MPB ratios were almost constant around a value of 2 (Figure 5), indicating that PPVMs appeared twice as bright as PMs. In women, similar conditions were found, as long as the collective contained no postmenopausal individuals. Once these were included, the collective’s mean additive MPPVB values climbed up to levels 2.5 times higher than corresponding MPB values and attained threefold higher MPPVB compared to MPB values as soon as over-79-year-old women were included.

These findings suggest that with the onset of menopause, the deterioration in women’s muscular resilience is mirrored by decreasing clustered mean ∆MVB − MPPVB values of ascendingly aged women (Figure 4b) and by increasing means of additive MPPVB/MPB ratios (Figure 5b), which was not found in men to a similar extent.

The method described in this article is resource-saving in clinical settings, as it only requires a lumbar MRI study of the index person. Measurements and calculations are performed quickly, deliver conclusive results, and allow clinicians to easily differentiate between healthy and pathologic muscle status. However, absolute figures might depend on the applied technical devices and processing software used in this investigation.

This study has several limitations. First, proposed parameters have not been validated in larger collectives yet. Second, T1-weighted axial MRI images are more specific for detecting fatty degeneration in muscles or vertebral bodies compared to T2-weighted images. However, in evaluated standard diagnostic lumbar MRI studies, axial image series were generally available as T2- rather than T1-weighted images. Third, applied single-slice measurements for quantifying sarcopenia were shown to be inferior compared to whole-body compartment investigations [26]. However, whole-body examinations are not yet practicable in clinical applications, as segmentation tasks and data processing are time-consuming and may require artificial intelligence [27]. An alternative method of assessing muscle quality by image pattern analysis might be texture analysis, which represents a promising muscle quality determining biomarker in the future [27].

## Figures and Tables

**Figure 2:**
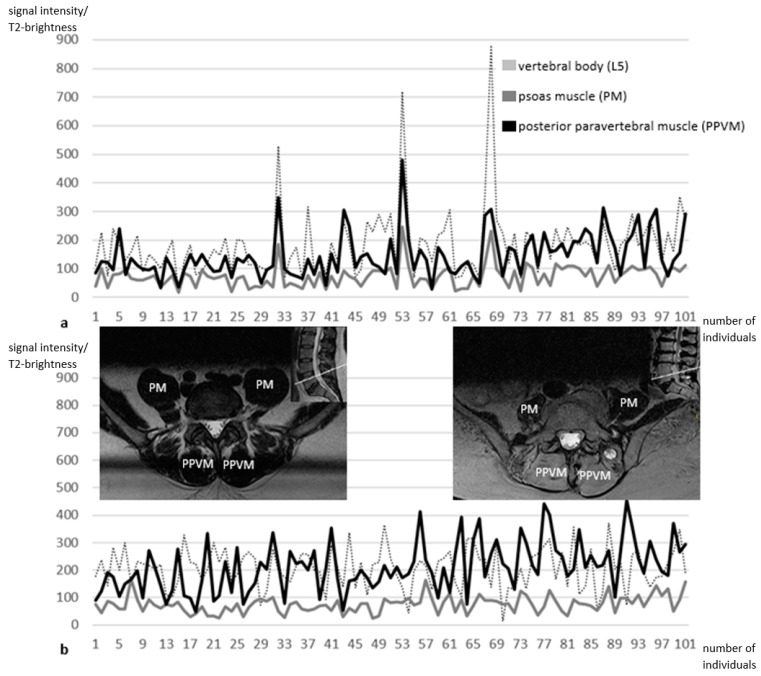
(**a**,**b**) Age-related brightness distributions in 101 men (**a**) and 101 women (**b**) from musculovertebral L5-level measurements in axial T2-weighted MRIs. PM brightness (gray curve) is constant in both graphs. In men, L5 brightness (dotted curve) is slightly higher than that of PPVMs (black curve). In aging women, PPVM brightness exceeds corresponding L5 brightness. Depicted T2-weighted axial MRI slices in the L5-level illustrate the age-related increasing brightness of PPVMs (left: a 41-year-old woman, right: an 85-year-old woman). L5 indicates lumbar vertebra V; MRI, magnetic resonance imaging; PM, psoas muscle; PPVM, posterior paravertebral muscle.

**Table 1:** Inclusion and exclusion criteria of the study.

Inclusion criteria	- Osseous mature individuals AND - Availability of lumbar MRI with axial T2 sequence showing the inferior lumbar levels, both psoas muscles, and both erector spinae muscles
Exclusion criteria	- Existence of synthetic implants in the lower lumbar spine OR - Condition after cement augmentation of the fifth lumbar vertebra OR - Evidence of the existence of tumorous lesions of paraspinal muscles or the fifth lumbar vertebra

To include an individual in the study, all inclusion criteria had to be fulfilled. For the exclusion of a subject, the presence of a single exclusion criterion sufficed. MRI indicates magnetic resonance imaging.

**Table 2:** Baseline characteristics. Significance was reached at *p* < 0.05; significant values are in bold.

Characteristic	Women (n = 101)	Men (n = 101)	*p*
Age (range), years	62.6 (34–85)	57.0 (21–90)	**0.009**
Prevalence of the diagnosis *…			
…fracture—n (%)	24 (23.8 **)	8 (7.9 **)	**0.002**
…spondylolisthesis—n (%)	15 (14.8 **)	5 (4.9 **)	**0.02**
…degenerative disc disease—n (%)	34 (33.7 **)	27 (26.7 **)	0.28
…disc herniation—n (%)	32 (31.7 **)	37 (36.6 **)	0.46
…spinal stenosis—n (%)	25 (24.7 **)	28 (27.7 **)	0.63
…previous lumbar fusion procedures—n (%)	7 (6.9 **)	5 (4.9 **)	0.55
…no relevant spinal alterations—n (%)	10 (9.9 **)	20 (19.8 **)	**0.048**

* Multiple diagnoses were possible. Therefore, the percentages do not add up to 100. ** Percentages are related to frequencies in groups “women” and “men”.

**Table 3:** Summary of results. Differences between groups were significant as to LIC area sizes and diameters of LV5, PMs, PPVMs, and MPPVB, but also as to ∆MVB − MPPVB and MPPVB/MPB ratio. Significance was reached at *p* < 0.05 (Student’s *t*-test); significant values are in bold.

Result	Women (n = 101)		Men (n = 101)		*p*
	Mean (Range)	95% CI	Mean (Range)	95% CI	
LIC area size of LV5s, mm^2^	881.0 (546.4–1460.8)	[844.9, 917.1]	1055.8 (199.3–1773.6)	[1011.7, 1099.9]	**<0.00001**
LIC longitudinal diameter of LV5, mm	33.3 (26.4–43.1)	[32.6, 34.0]	36.4 (15.9–47.5)	[35.6, 37.2]	**<0.00001**
LIC area size of PMs, mm^2^	697.3 (176.2–1475.3)	[651.9, 742.6]	1266.1 (420.6–2022.2)	[1195.7, 1336.5]	**<0.00001**
LIC transverse diameter of PM, mm	29.4 (15.0–43.3)	[28.4, 30.3]	39.7 (23.1–50.7)	[38.6, 40.9]	**<0.00001**
LIC area size of PPVMs, mm^2^	1004.4 (214.7–2398.0)	[934.5, 1074.3]	1347.8 (709.7–3129.4)	[1279.1, 1416.6]	**<0.00001**
LIC longitudinal diameter of PPVM, mm	35.2 (16.5–55.2)	[34.0, 36.4]	41.1 (30.1–63.1)	[40.1, 42.1]	**<0.00001**
MVB	207.4 (15.0–371.6)	[192.9, 221.8]	186.5 (20.3–880.8)	[163.3, 209.7]	0.14, n.s.
MPB	76.6 (24.5–167.2)	[70.6, 82.5]	74.1 (16.6–247.3)	[66.5, 81.6]	0.61, n.s.
MPPVB	210.3 (49.4–454.6)	[192.8, 227.8]	146.7 (28.2–479.7)	[131.6, 161.8]	**<0.00001**
∆MVB − MPPVB	−5.1 (−381.3–236.8)	[−27.6, 17.4]	39.3 (−108.4–573.1)	[22.0, 56.5]	**0.002**
MPPVB/MPB ratio	3.0 (1.0–10.4)	[2.7, 3.3]	2.2 (0.8–5.3)	[2.0, 2.4]	**<0.00001**

LIC indicates largest inscribed circle; LV5, lumbar vertebra V; PM, psoas muscle; PPVM, posterior paravertebral muscle; mm, millimeter; MVB, mean vertebral (body) brightness; MPB, mean psoas (muscle) brightness; MPPVB, mean posterior paravertebral (muscle) brightness; ∆MVB − MPPVB, difference between mean vertebral brightness and mean posterior paravertebral (muscle) brightness; CI, confidence interval; n.s., not significant.

## Data Availability

The data presented in this study are available within the article and its Supplementary Materials.

## Supplementary Materials

The following supporting information can be downloaded at: https://www.mdpi.com/article/10.3390/jcm13237233/s1, Table S1: Supplemental Raw Data Table.
