# Supplementary material for: Lumbar Magnetic Resonance Imaging Shows Sex-Specific Alterations During Musculoskeletal Aging—A Radio-Anatomic Investigation Involving 202 Individuals
Source: J Clin Med. 2024 Nov 28;13(23):7233. doi: 10.3390/jcm13237233 (PMC11642922; doi:10.3390/jcm13237233)
Supplement: Supplementary file 1 [file jcm-13-07233-s001.zip › jcm-3315676-supplementary.pdf]

Supplementary table. Raw data (age, measured mean inner circle brightness values of vertebral bodies (MVB), psoas muscles (MPB), and posterior paravertebral muscles (MPPVB)), including calculated differences between MVB and MPPVB (ΔMVB − MPPVB) and means of cumulated ΔMVB − MPPVB values up to the indexed age

Age (women)	MVB	MPB	MPPVB	ΔMVB − MPPVB	means of cumulated Δ	Age (men)	MVB	MPB	MPPVB	ΔMVB − MPPVB	means of cumulated Δ
34	178.7	74.5	90.7	88.0	n.a.	21	112.1	38.3	85.3	26.8	n.a.
34	236.4	46.0	125.0	111.5	99.73	24	225.5	102.1	125.4	100.2	63.48
35	142.8	88.5	192.1	−49.3	50.05	30	74.0	33.1	124.1	−50.1	25.63
36	282.0	77.3	176.0	106.0	64.04	30	241.3	79.3	94.7	146.7	55.89
38	201.9	58.1	102.9	99.0	71.03	32	182.2	80.9	240.3	−58.1	33.10
39	298.9	59.2	149.1	149.9	84.17	32	132.2	95.9	80.7	51.5	36.17
39	148.2	112.6	167.2	−19.0	69.43	32	158.1	66.3	135.8	22.3	34.19
39	230.6	93.9	196.9	33.8	64.97	33	215.0	60.7	112.7	102.3	42.70
39	222.0	49.3	99.8	122.3	71.33	34	87.8	60.5	98.7	−10.9	36.75
39	217.7	92.8	271.0	−53.3	58.87	35	151.3	68.5	94.7	56.6	38.74
40	202.8	73.5	213.5	−10.7	52.55	35	130.4	75.4	107.1	23.4	37.34
40	219.8	60.6	144.5	75.3	54.44	37	100.3	35.2	33.7	66.6	39.78
41	111.1	76.5	75.9	35.3	52.97	38	147.5	55.1	140.2	7.3	37.28
42	101.8	71.5	126.2	−24.4	47.44	38	199.7	78.0	97.5	102.2	41.91
45	159.0	83.0	278.1	−119.1	36.34	38	20.3	16.6	36.6	−16.3	38.03
45	328.0	53.9	105.8	222.3	47.96	41	125.3	85.5	95.3	30.0	37.53
46	227.8	30.2	98.3	129.5	52.75	42	182.8	73.0	150.5	32.4	37.23
47	223.1	41.3	49.4	173.8	59.48	42	78.7	34.1	112.5	−33.8	33.28
47	164.4	67.0	164.8	−0.3	56.33	43	92.3	95.3	149.7	−57.4	28.51
47	203.0	32.1	333.8	−130.8	46.97	44	121.8	73.2	116.7	5.1	27.34
47	299.2	32.5	87.2	212.1	54.83	44	167.4	67.0	90.5	76.9	29.70
48	228.1	24.5	106.3	121.9	57.88	44	146.9	72.5	94.3	52.7	30.75
49	281.5	68.0	232.2	49.4	57.51	45	207.3	78.8	144.8	62.6	32.13
49	157.6	50.2	118.6	39.1	56.74	46	92.0	19.8	67.4	24.7	31.82
50	184.2	79.1	281.6	−97.4	50.57	49	200.9	66.0	137.2	63.7	33.09
51	250.1	31.5	75.4	174.7	55.35	49	195.4	74.2	122.1	73.4	34.64
52	266.9	65.4	120.6	146.4	58.72	50	111.0	27.9	147.6	−36.6	32.00

Age (women)	MVB	MPB	MPPVB	ΔMVB − MPPVB	means of cumulated Δ	Age (men)	MVB	MPB	MPPVB	ΔMVB − MPPVB	means of cumulated Δ
52	243.9	86.5	151.8	92.2	59.91	51	118.3	39.8	119.6	−1.3	30.81
52	73.9	95.7	229.6	−155.7	52.48	51	103.4	34.6	49.5	53.9	31.61
52	124.4	86.2	204.8	−80.4	48.05	51	95.1	57.2	96.7	−1.6	30.50
53	276.9	102.6	336.5	−59.6	44.58	51	112.5	35.5	108.9	3.7	29.64
53	197.4	51.3	223.0	−25.6	42.38	52	529.1	186.3	348.3	180.9	34.36
53	176.2	28.9	78.6	97.7	44.06	52	69.6	36.2	98.9	−29.3	32.43
54	158.7	75.7	267.7	−109.0	39.56	52	135.5	49.8	82.9	52.7	33.03
54	214.1	85.4	222.7	−8.6	38.18	52	173.7	41.2	74.9	98.9	34.91
54	260.9	59.4	231.7	29.3	37.94	52	58.2	29.8	65.2	−7.0	33.74
55	258.6	54.4	199.5	59.1	38.51	53	316.2	76.2	134.9	181.4	37.73
55	201.9	59.8	271.0	−69.1	35.68	53	84.3	35.3	80.6	3.8	36.84
56	195.2	70.0	93.1	102.2	37.38	54	150.9	88.1	142.9	8.0	36.10
56	91.6	73.0	207.7	−116.1	33.55	54	78.7	26.9	41.3	37.4	36.13
57	291.8	54.3	354.3	−62.5	31.20	54	190.9	72.8	154.3	36.6	36.14
58	211.3	90.1	170.8	40.6	31.43	54	125.0	35.9	88.4	36.7	36.15
58	137.7	30.8	52.8	85.0	32.67	54	264.2	93.9	306.9	−42.7	34.32
62	337.6	62.1	159.9	177.7	35.97	54	192.7	74.8	249.4	−56.7	32.25
62	136.4	47.8	167.4	−31.0	34.48	55	71.5	63.8	103.0	−31.5	30.84
62	228.7	79.2	201.1	27.7	34.33	55	99.2	38.5	141.0	−41.8	29.26
63	109.1	80.0	172.8	−63.7	32.25	55	264.2	72.5	153.1	111.1	31.00
63	220.2	24.9	134.9	85.3	33.35	55	230.2	92.5	118.8	111.5	32.68
63	228.5	32.4	157.1	71.4	34.13	55	289.0	93.5	107.6	181.5	35.71
64	365.1	96.7	218.8	146.4	36.37	56	229.2	88.0	81.6	147.7	37.95
64	242.3	81.4	171.9	70.5	37.04	57	292.8	103.2	204.1	88.8	38.95
64	180.4	83.8	212.6	−32.2	35.71	57	97.0	30.5	82.0	15.1	38.49
66	138.0	82.0	173.4	−35.4	34.37	57	718.7	247.3	479.7	239.1	42.27
66	43.7	99.6	187.4	−143.7	31.07	59	250.3	108.1	200.9	49.5	42.40
67	199.0	73.9	233.2	−34.2	29.89	59	68.7	35.9	95.8	−27.1	41.14
67	236.0	80.9	413.1	−177.1	26.19	59	208.3	66.8	166.9	41.4	41.15
67	218.4	162.9	236.8	−18.4	25.41	59	194.0	63.5	130.4	63.6	41.54

Age (women)	MVB	MPB	MPPVB	ΔMVB − MPPVB	means of cumulated Δ	Age (men)	MVB	MPB	MPPVB	ΔMVB − MPPVB	means of cumulated Δ
68	129.4	95.1	188.4	−59.0	23.95	59	126.1	31.5	28.2	98.0	42.51
68	231.0	35.8	99.6	131.4	25.77	60	219.6	72.3	174.3	45.4	42.56
69	242.9	84.7	210.5	32.4	25.89	60	228.3	93.1	145.2	83.1	43.24
69	269.7	116.3	118.1	151.7	27.95	61	305.3	104.0	90.3	215.0	46.05
70	154.9	47.9	257.2	−102.3	25.85	61	69.8	21.3	83.6	−13.8	45.09
70	103.8	93.7	394.0	−290.2	20.83	61	73.8	31.2	108.5	−34.7	43.82
70	312.9	34.2	76.1	236.8	24.20	61	119.3	31.3	127.1	−7.8	43.02
70	317.2	69.5	308.5	8.7	23.97	62	117.3	73.0	80.6	36.8	42.92
70	237.7	113.0	388.7	−151.0	21.32	62	67.2	38.4	50.3	16.9	42.53
71	243.9	90.3	174.2	69.7	22.04	64	351.2	116.9	285.3	66.0	42.87
72	136.9	89.1	260.0	−123.1	19.90	64	880.8	232.3	307.7	573.1	50.67
73	302.0	86.6	310.1	−8.1	19.50	64	270.2	99.8	196.3	74.0	51.01
74	15.0	76.4	222.0	−207.0	16.26	64	223.4	71.4	74.8	148.7	52.40
74	134.2	77.9	205.9	−71.7	15.02	64	120.5	32.9	173.9	−53.4	50.91
74	132.0	43.8	129.9	2.1	14.85	64	223.6	93.7	164.8	58.9	51.02
75	207.1	122.7	353.3	−146.2	12.64	65	65.9	23.3	78.7	−12.8	50.15
75	132.1	111.2	299.1	−167.0	10.21	65	230.3	120.7	178.0	52.3	50.18
75	249.7	73.0	218.9	30.8	10.49	67	204.4	103.9	219.5	−15.1	49.31
76	260.6	35.0	184.7	76.0	11.35	67	83.9	43.4	109.4	−25.5	48.33
78	289.5	67.5	440.7	−151.2	9.24	67	235.7	82.6	225.9	9.8	47.83
78	314.4	127.2	401.6	−87.2	8.00	68	134.4	42.5	159.4	−25.0	46.89
78	166.7	88.3	272.6	−105.9	6.56	69	240.6	118.1	164.5	76.2	47.26
79	262.2	51.2	252.7	9.5	6.60	69	171.9	99.0	188.0	−16.1	46.47
79	141.6	34.4	179.0	−37.4	6.05	70	244.8	109.3	141.9	102.9	47.17
79	356.2	92.3	202.2	154.0	7.86	72	191.8	109.6	196.5	−4.6	46.54
79	112.1	78.3	347.6	−235.5	4.93	72	184.3	102.1	194.2	−9.9	45.86
80	140.8	74.7	209.5	−68.7	4.05	73	193.9	74.7	240.1	−46.2	44.76
80	277.4	71.5	253.7	23.7	4.28	73	181.3	101.9	221.1	−39.8	43.77
80	59.6	52.9	211.9	−152.3	2.46	74	113.7	38.5	120.7	−7.0	43.18
81	115.7	82.9	217.1	−101.4	1.27	75	258.5	77.2	314.2	−55.7	42.04

Age (women)	MVB	MPB	MPPVB	ΔMVB − MPPVB	means of cumulated Δ	Age (men)	MVB	MPB	MPPVB	ΔMVB − MPPVB	means of cumulated Δ
81	371.6	141.6	272.2	99.5	2.38	75	172.1	112.7	231.3	−59.2	40.89
81	202.7	45.0	102.4	100.3	3.48	75	92.7	51.0	171.0	−78.3	39.55
81	231.6	99.9	264.8	−33.2	3.08	75	182.4	75.9	75.5	107.0	40.30
81	73.2	98.8	454.6	−381.4	-1.15	75	208.4	92.6	192.0	16.4	40.04
81	258.7	79.3	360.6	−101.9	-2.24	76	291.0	108.7	217.6	73.5	40.40
81	272.1	108.8	243.2	29.0	-1.91	78	181.2	96.6	289.7	−108.5	38.80
82	190.7	68.6	189.9	0.8	-1.88	79	222.6	97.5	102.9	119.7	39.66
82	138.7	104.5	304.5	−165.8	-3.60	80	269.2	107.7	264.1	5.1	39.30
82	175.1	143.5	237.6	−62.5	-4.22	81	199.9	82.6	308.4	−108.5	37.76
83	176.8	108.5	196.0	−19.2	-4.37	81	118.2	37.5	131.2	−13.0	37.23
85	226.5	132.0	181.2	45.4	-3.86	81	225.5	92.2	73.8	151.8	38.40
85	264.5	51.7	372.0	−107.5	-4.91	84	158.7	104.8	128.6	30.2	38.32
85	348.6	92.8	267.1	81.6	-4.05	87	351.9	91.2	155.2	196.7	39.90
85	186.4	157.2	294.4	−108.0	-5.08	90	267.8	112.5	291.1	−23.3	39.28

MVB indicates mean vertebral (body) brightness; MPB, mean psoas (muscle) brightness; MPPVB, mean posterior paravertebral (muscle) brightness; ΔMVB − MPPVB, difference between mean vertebral brightness and mean posterior paravertebral (muscle) brightness; n.a., not applicable.
